# Mouse Hepatitis Coronavirus RNA Replication Depends on GBF1-Mediated ARF1 Activation

**DOI:** 10.1371/journal.ppat.1000088

**Published:** 2008-06-13

**Authors:** Monique H. Verheije, Matthijs Raaben, Muriel Mari, Eddie G. te Lintelo, Fulvio Reggiori, Frank J. M. van Kuppeveld, Peter J. M. Rottier, Cornelis A. M. de Haan

**Affiliations:** 1 Virology Division, Department of Infectious Diseases and Immunology, Utrecht University, Utrecht, The Netherlands; 2 Department of Cell Biology and Institute of Biomembranes, University Medical Centre Utrecht, Utrecht, The Netherlands; 3 Department of Medical Microbiology, Radboud University Nijmegen Medical Centre, Nijmegen Centre for Molecular Life Sciences, Nijmegen, The Netherlands; University of North Carolina, United States of America

## Abstract

Coronaviruses induce in infected cells the formation of double membrane vesicles, which are the sites of RNA replication. Not much is known about the formation of these vesicles, although recent observations indicate an important role for the endoplasmic reticulum in the formation of the mouse hepatitis coronavirus (MHV) replication complexes (RCs). We now show that MHV replication is sensitive to brefeldin A (BFA). Consistently, expression of a dominant-negative mutant of ARF1, known to mimic the action of the drug, inhibited MHV infection profoundly. Immunofluorescence analysis and quantitative electron microscopy demonstrated that BFA did not block the formation of RCs *per se*, but rather reduced their number. MHV RNA replication was not sensitive to BFA in MDCK cells, which are known to express the BFA-resistant guanine nucleotide exchange factor GBF1. Accordingly, individual knockdown of the Golgi-resident targets of BFA by transfection of small interfering RNAs (siRNAs) showed that GBF1, but not BIG1 or BIG2, was critically involved in MHV RNA replication. ARF1, the cellular effector of GBF1, also appeared to be involved in MHV replication, as siRNAs targeting this small GTPase inhibited MHV infection significantly. Collectively, our results demonstrate that GBF1-mediated ARF1 activation is required for efficient MHV RNA replication and reveal that the early secretory pathway and MHV replication complex formation are closely connected.

## Author Summary

Coronaviruses are the causative agents of many respiratory and enteric infections in humans and animals. As with all viruses, virtually all of the steps of their infection cycle depend on host cellular factors. As the first and most crucial step after their entry into cells, coronaviruses assemble their replication complexes (RCs) in association with characteristic, newly induced membranous structures. The cellular pathways hijacked by these plus-strand RNA viruses to create these “factories” have not been elucidated. Here, we study the involvement of the secretory pathway in mouse hepatitis coronavirus (MHV) replication by using the drug brefeldin A (BFA), which is known to interfere with ER–Golgi membrane traffic by inhibiting the activation of ADP-ribosylation factor (ARF) small GTPases. Our observations show that MHV RNA replication is sensitive to BFA. In agreement herewith we demonstrate, by using various techniques, that the BFA-sensitive guanidine nucleotide exchange factor GBF1 and its downstream effector ARF1 are of critical importance for coronavirus replication. From our results we conclude that MHV RNA replication depends on GBF1-mediated ARF1 activation. Our study provides new insights into the close connection between MHV replication and the early secretory pathway.

## Introduction

Viruses rely on cellular host factors for virtually all steps of their infection cycle. However, the cellular proteins required and the cellular pathways hijacked by viruses have hardly been elucidated. All positive-strand RNA viruses assemble in infected cells their replication complexes (RCs) in association with intracellular membranes [1],[2],[3],[4],[5]. The induction of such local micro-environments is likely advantageous for the virus, as membrane association may facilitate the recruitment of both the viral and cellular components involved in RNA replication. Alternatively, membrane association may provide a shielded environment that prevents the activation of, or protects against, antiviral host cell responses like those mediated by interferon.

Coronaviruses belong to a family of enveloped positive-strand RNA viruses in the order *Nidovirales*. Upon translation of the viral genomic RNA, two very large polyproteins (approximately 4,000 and 7,000 amino acids) are synthesized, the autoproteolytic cleavage products of which collectively form the RCs. These RCs are associated with double membrane vesicles (DMVs [6],[7],[8]), which appear as cytoplasmic foci when analyzed by fluorescence light microscopy and increase in number during the course of the infection [6],[8],[9],[10]. It is plausible that the non-structural viral proteins (nsps) mediate the formation of DMVs by modifying intracellular membranes and by recruiting cellular components to their need. Recent studies suggest the endoplasmic reticulum (ER) to be the lipid donor compartment of the membrane-bound coronavirus RCs [10],[11],[12],[13], although co-localization of nsps with markers for endosomes, Golgi and autophagosomes has also been described [7],[10],[14],[15],[16].

Brefeldin A (BFA) is a well known fungal metabolite that induces the redistribution of Golgi proteins into the ER [17],[18], effectively resulting in the block of transport though the secretory pathway [19],[20]. This drug inhibits the activation of ADP-ribosylation factor (ARF) small GTPases by targeting the large guanine nucleotide exchange factors (GEFs) GBF1 (Golgi-specific resistance factor 1), and BIG (BFA-inhibited GEF) 1 and 2 [21],[22],[23]. More specifically, BFA locks ARF*GDP when bound to GEF, thereby blocking the GEF activity at an early stage of the reaction, prior to guanine nucleotide release [24],[25]. The large GEFs function in the ER to Golgi transport pathway [26] and localize to the cis-(GBF1) and trans-sides (BIG1 and BIG2) of the Golgi complex [27]. The cellular effectors of these GEFs, ARFs, are divided into three classes: Class I (ARF1-3), Class II (ARF 4 and 5), and Class III (ARF6) [28]. Class I ARFs regulate the assembly of coat complexes onto vesicles budding from compartments along the secretory pathway and activate lipid-modifying enzymes (reviewed in [29],[30]). While the function of Class II ARFs remains largely unclear, the Class III ARF6 is thought to regulate endosomal membrane traffic [31],[32]. GBF1 and the BIGs are likely to activate distinct subclasses of ARFs at specific locations in order to regulate different types of transport routes [27].

In the field of virology, BFA has been used, besides for studying viral protein transport and virus assembly [33],[34],[35],[36],[37],[38], to investigate the formation of RCs and RNA replication of several positive-strand RNA viruses [39],[40],[41],[42]. For example, poliovirus RNA replication was shown to be sensitive to BFA. In the presence of this drug, poliovirus replication sites were not formed and RNA replication was completely blocked [41],[43]. Remarkably, other members of the picornavirus family appeared to differ in their sensitivity to BFA. Whereas echovirus 11 RNA replication was strongly inhibited by BFA, RNA replication of encephalomyocarditis virus was not affected at all, while parechovirus 1 exhibited an intermediate sensitivity to it [44].

Relatively little is known about the host pathways involved in coronavirus RNA replication and in RC formation. Recently, we demonstrated the important role of the ER in the generation of the RCs. While MHV nsp4 was localized to this organelle when expressed alone, it was recruited to the replication complexes in infected cells [11]. Furthermore, coronaviral replication was inhibited when the ER export machinery was blocked by use of the kinase inhibitor H89 or by expression of a dominant active mutant of Sar1 [11]. Other cellular proteins and pathways are likely to contribute to the formation of the coronavirus RCs as well. Here, we studied the involvement of BFA-sensitive pathways in MHV replication and RC formation. Our results demonstrate that GBF1-mediated ARF1 activation is required for efficient MHV RNA replication. Moreover, together with our recent observation about the relevance of the ER in the same process, our data reveal that the early secretory pathway and MHV replication are intimately connected.

## Results

### MHV genomic RNA replication is sensitive to BFA

**Figure 1:**
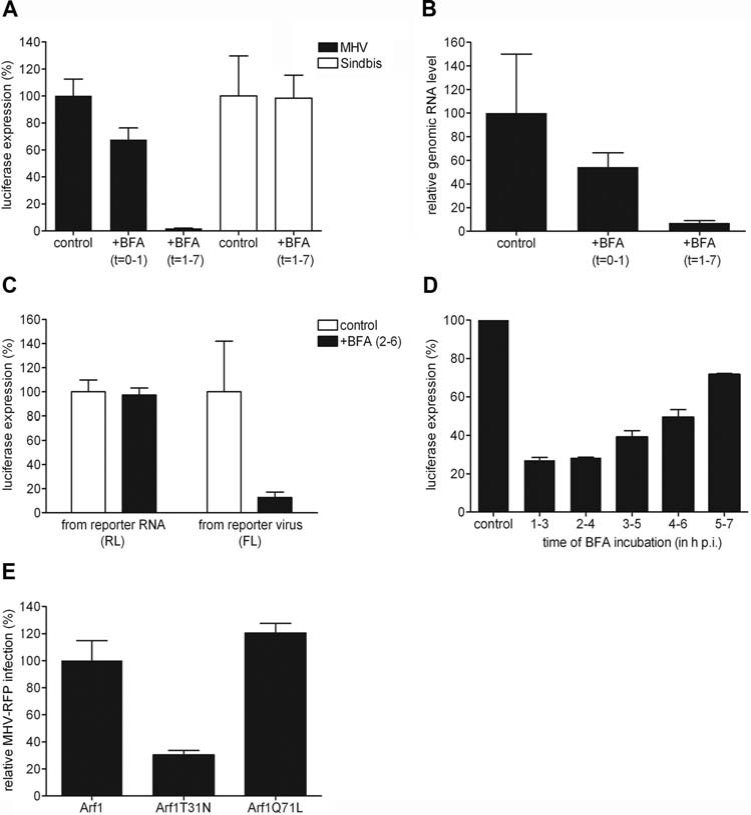
BFA inhibits MHV replication in mouse LR7 cells. (A, B, D) LR7 cells were inoculated with MHV-EFLM or with Sindbis pseudovirus particles containing a luciferase replicon and incubated with 5 µg/ml BFA during the indicated time periods. At the end of each incubation period, virus replication was analyzed by determining the luciferase expression level (A and D) or the amount of viral genomic RNA (B) as described in the Material and Methods. (C) LR7 cells were inoculated with MHV-2aFLS, transfected with synthetic RNA transcribed from pM5f-RL-M3, and incubated from 2–6 h p.i. in the presence or absence of 5 µg/ml BFA. *Renilla* (RL) and firefly (FL) luciferase expression levels were determined in the cell lysates at 6 h p.i. and are depicted relative to untreated samples; (E) LR7 cells were transfected with pARF1-YFP, pARF1T31N-YFP, or pARF1Q71L-YFP and inoculated with MHV-RFP (moi of 1) 24 h later. At 18 h p.i. FACS analyses were performed as described in Materials and Methods. The percentages of GFP/YFP positive cells that were also RFP positive were determined relative to wild type ARF1 expressing cells. The results of representative experiments performed in triplicate are shown. Error bars indicate standard deviations.

BFA is known to disturb membrane traffic in most cell types, resulting in a redistribution of Golgi proteins into the ER [17],[18]. We first confirmed the sensitivity of murine LR7 cells to BFA by immunofluorescence using antibodies directed against the Golgi protein marker GM130 [45]. Indeed, after treatment of the cells with 5 µg/ml BFA for 1 h, the typical Golgi staining pattern of GM130 was lost, concomitant with a reticular redistribution of the protein marker (data not shown). Next, we tested whether MHV infection was sensitive to BFA. Therefore, LR7 cells were inoculated with a luciferase-expressing recombinant of MHV-A59 (MHV-EFLM) in the presence or absence of 5 µg/ml BFA. After 1 h, the inoculum was removed and the cells were further incubated either in the presence or in the absence of BFA. At 7 h p.i., the intracellular luciferase expression level was determined relative to untreated cells. Luciferase expression was inhibited more than 95% when BFA was present from 1–7 h p.i., whereas BFA treatment during virus inoculation had only a minor effect on reporter gene expression (Fig. 1A). Although this latter decrease might have resulted in part from a reduced entry, the negative effect of BFA on MHV replication and transcription is evident from the profoundly impaired MHV reporter gene expression when BFA was added post inoculation (1–7 h p.i.).

In a control experiment, the effect of BFA on Sindbis virus replication in LR7 cells was assayed by using Sindbis pseudovirus particles containing luciferase-expressing replicons. As described previously [46], Sindbis virus replication was not affected by the BFA treatment (Fig. 1A). This result indicates that the observed effect of BFA on MHV-driven luciferase expression was not due to non-specific drug-induced toxicity.

Although we have demonstrated in previous studies that reporter gene expression by MHV is a reliable measure for coronavirus replication [47], we wanted to confirm that the reduction in luciferase expression resulted from a corresponding decrease in viral RNA synthesis rather than from inhibition of viral protein translation. To this end, a similar experiment as shown in Fig. 1A was performed, in which the amount of intracellular genomic viral RNA was determined by real-time Taqman PCR. As for the luciferase expression levels, the amount of genomic RNA was found to be severely reduced when BFA was added directly after the virus inoculation (Fig. 1B), whereas a less profound effect was observed when cells were treated during virus inoculation. Very similar results were obtained when targeting the Taqman PCR to a different region of the viral genome (data not shown). To more directly check for an effect of BFA on the translation of viral mRNAs, we performed an additional experiment. LR7 cells were infected at high multiplicity with the recombinant virus MHV-2aFLS, which expresses the firefly luciferase, and subsequently transfected with a synthetic mRNA encoding *Renilla* luciferase. This synthetic mRNA mimics viral mRNAs as it contains 5' and 3' untranslated regions identical to those found in the viral genome. The cells were incubated in the presence or absence of BFA (2–6 h p.i.) after which the intracellular *Renilla* and firefly luciferase expression levels were determined. The results show that BFA treatment did not inhibit the synthesis of *Renilla* luciferase from the synthetic mRNA, while firefly luciferase expression driven by the recombinant virus was severely affected (Fig. 1C). *Renilla* luciferase expression was also not affected in the absence of a viral infection (data not shown). All together, these results indicate that BFA inhibits MHV RNA replication while translation of viral mRNAs is not affected.

Next, we determined the post inoculation period during which MHV replication was most sensitive to BFA, by analyzing the luciferase expression levels as they are a reliable measure for RNA replication. Thus LR7 cells infected with MHV-EFLM were treated with BFA for overlapping 2 h periods. At the end of each incubation period the intracellular luciferase expression levels were determined and compared to those in mock-treated cells. The results showed that replication was affected throughout the course of the infection (Fig. 1D); however, the effects were most pronounced during the early phases of infection.

### ARF1-T31N inhibits MHV replication

To confirm our observation that BFA inhibits MHV replication but also to prove that the effects of this drug are due to the inhibition of GEF activities, we next analyzed to what extent the expression of a dominant-negative mutant of ARF1 (T31N) would affect MHV infection. This ARF1 mutant has a decreased affinity for GTP and, following GDP displacement, it remains ‘nucleotide-free’ for a longer period than wt ARF1 [48]. As a consequence, expression of ARF1-T31N mirrors the effects of BFA [49]. In addition to this protein, we included a constitutive-active ARF1 mutant (ARF1-Q71L), which persists in the GTP-bound state longer than wild-type ARF, resulting in a prolonged ARF1 activation. Expression of this latter mutant is known to inhibit transport at later steps in the secretory pathway, e.g. from vesicular tubular clusters (VTC) to the Golgi complex and between Golgi stacks [49]. LR7 cells were transfected with plasmids expressing YFP fusions of either wild type ARF1, ARF1-T31N or ARF1-Q71L. After transfection, the cells were inoculated with an RFP-expressing MHV-A59 recombinant (MHV-RFP) that allows flow cytometric analysis of MHV replication [11]. The percentage of RFP-positive cells in the YFP-expressing population was determined relative to that of the wild type ARF1 expressing cells (Fig. 1E). Overexpression of the wt ARF1 fusion protein itself did not significantly affect MHV infection when compared to non-transfected cells (data not shown). The results indicate that over-expression of the dominant-negative ARF1 mutant inhibited MHV infection profoundly, thereby confirming the results obtained with BFA. In contrast, expression of the constitutive-active mutant of ARF1 did not influence MHV replication.

### BFA inhibits but does not entirely block the formation of MHV RCs

**Figure 2:**
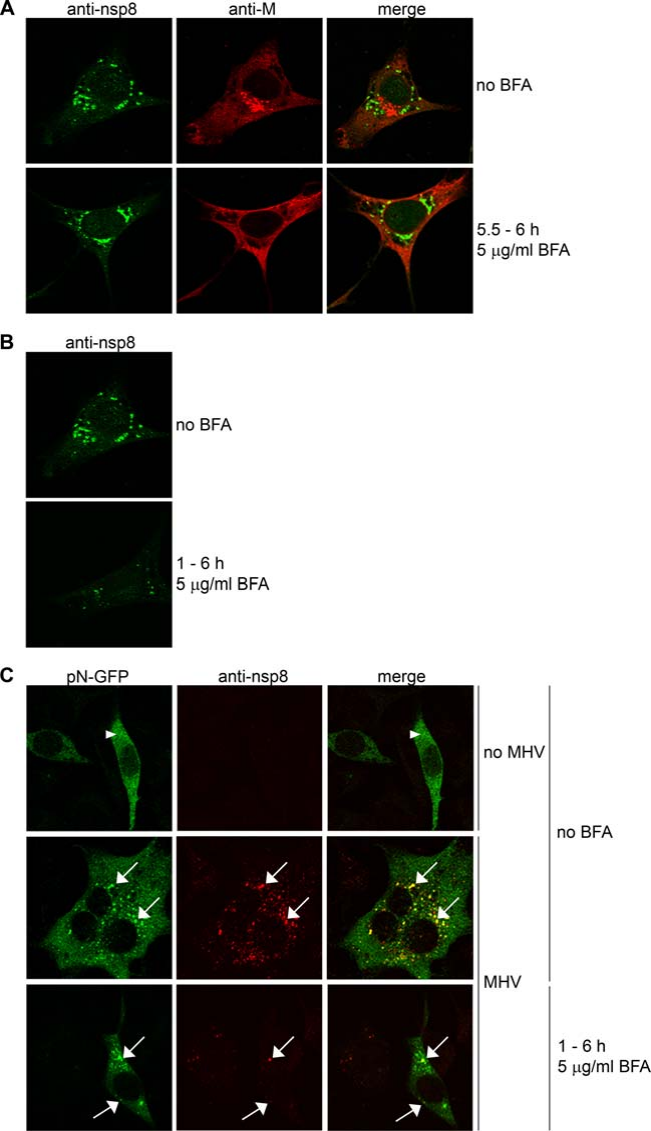
Immunofluorescence analysis of MHV RCs. LR7 cells were inoculated with MHV-A59 and subsequently mock-treated (panel A, upper row), treated with 5 µg/ml BFA from 5.5–6 h p.i. (panel A, lower row) or from 1–6 h p.i. (panel B). Immunostaining was performed using antibodies against nsp8 (anti-nsp8) and against the M protein (anti-M). LR7 cells were transfected with pN-EGFP and subsequently mock-infected (panel C, upper row), infected with MHV-A59 (panel C, middle row), or infected with MHV-A59 and treated with 5 µg/ml BFA from 1 to 7 h p.i. (panel C, bottom row). At 7 h p.i., cells were fixed and an immunostaining was performed using the nsp8 antibodies. Identical confocal microscopy settings were used for mock-treated and BFA-treated samples. Arrowheads in panel C indicate cytosolic staining; arrows indicate nsp8-positive foci.

As BFA is known to affect intracellular vesicle formation and transport, and because MHV replicates its genome in association with DMVs, we next investigated the effect of BFA on the assembly of the MHV RCs. First, we checked whether the morphological integrity of the RCs was affected in the presence of BFA. Therefore, LR7 cells infected with MHV-A59 were treated with BFA for 30 minutes starting 5.5 h p.i. They were subsequently fixed and processed for immunofluorescence using antibodies both against nsp8, which served as a protein marker for the MHV replication sites [50],[51], and against the viral structural protein M, known to reside in the Golgi [52]. The nsp8 antibody revealed the typical perinuclear staining pattern in both treated and non treated infected cells (Fig. 2A). In contrast, a dispersed distribution of M protein was observed in BFA-treated cells reflecting the collapse of the Golgi, whereas in non-treated cells the M protein showed a clear Golgi-like staining (Fig. 2A). These results indicate that, once formed, the replication sites are not disrupted by BFA.

Subsequently, we investigated whether BFA inhibited RC formation early in the infection. BFA was therefore added to LR7 cells directly after inoculation with MHV-A59 and staining was performed at 6 h p.i using the nsp8 antibody. Although some perinuclear staining of nsp8 could be detected in BFA-treated cells, the number and intensity of the nsp8 containing foci were clearly reduced when compared to non-treated cells (Fig. 2B). We next investigated whether these nsp8 puncta represented MHV replication sites. Therefore, we studied the ability of the nsp8 foci to recruit the nucleocapsid protein N, a protein previously shown to localize to the RCs [9],[50]. Three parallel cultures of LR7 cells were transfected with a plasmid coding for a MHV N-GFP fusion protein and 24 h post transfection two of them were infected with MHV-A59. BFA (5 µg/ml) was added to one of these latter cultures directly after inoculation (t = 1 h p.i.). At 6 h p.i., the cells were fixed and subsequently processed for immunofluorescence using the anti-nsp8 antibody (Fig. 2C). As expected, N-GFP was diffusely localized to the cytosol in non-infected cells (indicated by an arrowhead in Fig. 2C). In contrast, when cells were infected with MHV, this fusion protein also appeared in foci that co-localized with nsp8 (indicated by arrows in Fig. 2C). This co-localization was observed both in mock- and in BFA-treated cells, indicating that the nsp8 foci that had been formed in the presence of BFA, though decreased in number and intensity, correspond with the replication sites. In complete agreement with the luciferase expression data shown above, this result demonstrates that BFA inhibits, but does not completely block, the formation of RCs.

### BFA treatment reduces the number of DMVs

**Figure 3:**
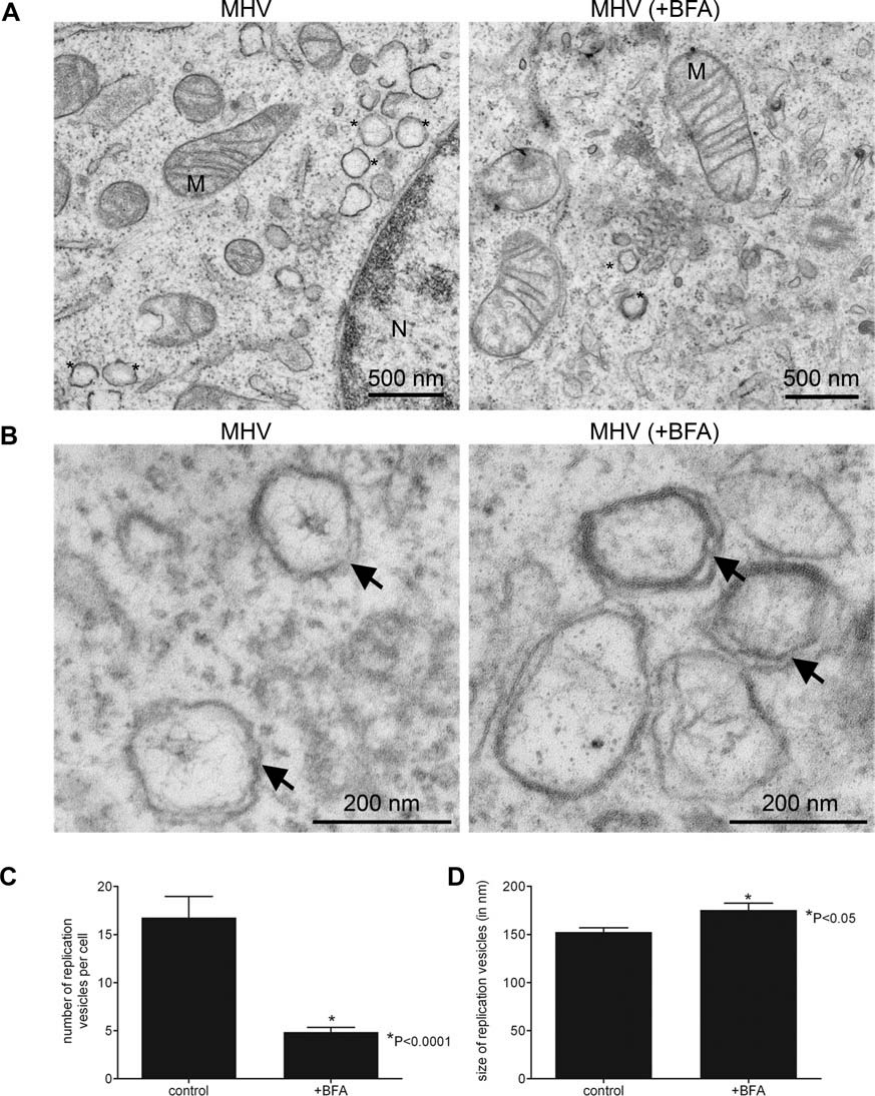
Ultrastructural analysis of MHV-infected LR7 cells. LR7 cells were inoculated with MHV-A59 and treated with or without 5 µg/ml BFA from 1–6 h p.i, chemically fixed and embedded with Epon resin. (A) Numerous clusters of virus-induced DMVs (indicated by *) were found in the perinuclear region of the cell (N-nucleus; M-mitochondrion); Panel B shows a close view of DMVs, clearly demonstrating the presence of double membranes (indicated by arrows); (C) The average number of DMVs per cell obtained by counting 20 infected cells; (D) Average DMV diameter obtained measuring 38 of them. Error bars indicate standard error of the mean (SEM).

To study the effects of BFA on the DMVs at an ultrastructural level, MHV-infected LR7 cells were fixed at 6 h p.i. and embedded in Epon resin in order to be analyzed by electron microscopy. DMVs (indicated by the asterisks in Fig. 3A) were always seen organized in clusters often located in the perinuclear area. The morphology and dimensions of these vesicles were similar to those previously described for the DMVs harboring the RCs [7],[8],[10],[12],[14],[53]. Importantly, these vesicles were not observed in mock-infected cells (data not shown). Fig. 3B shows a close view of these DMVs, in which the translucent interior is surrounded by a double membrane. The presence of an inner web-like structure is most likely artificial [10].

Treatment of cells with BFA (1–6 h) led to the expected disappearance of an apparent Golgi complex with the concomitant expansion of the ER volume (not shown). In these cells, vesicles with a morphology almost identical to those present in non BFA-treated cells were observed (Fig. 3A). However, the number of these DMVs was significantly decreased (p<0.005) in BFA-treated cells as compared to non-treated cells (4.9 vs. 16.8 on average per section, Fig. 3C). The reduction in the number of DMVs is likely to be an underestimation as only EM sections were included in the analyses in which at least one replication vesicle could be detected. Strikingly, the double membrane of the replication vesicles was visually more pronounced in BFA-treated cells than in untreated cells (Fig. 3B), which might relate to the swelling of the ER observed after BFA addition. The DMVs were slightly bigger in the BFA-treated cells (175.4 nm +/− 7.1 compared to 152.4 nm +/− 4.5 in non-treated cells; p<0.05; Fig. 3D), although the significance of this latter observation is not clear at present.

Overall, our ultrastructural analysis of MHV-infected cells confirms that treatment of cells with BFA decreased the number of replication vesicles, consistent with the reduced viral RNA replication in the presence of BFA.

### The GEF GBF1 is required for MHV replication

**Figure 4:**
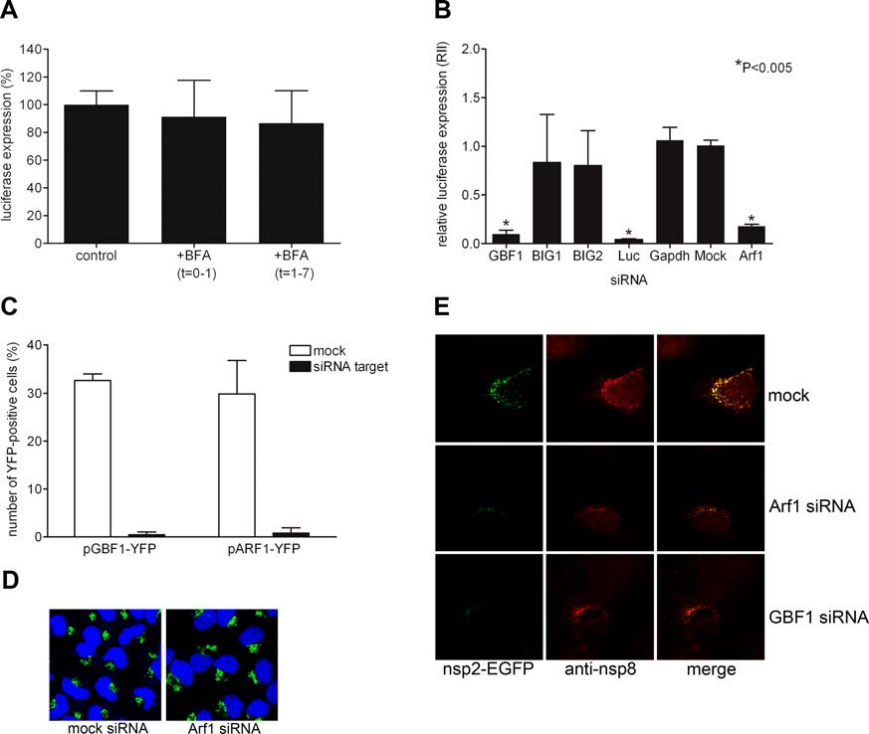
The role of Golgi-residing GEFs in MHV replication. (A) MDCK(MHVR) cells were inoculated with MHV-EFLM and incubated with 5 µg/ml BFA during the indicated time periods. At 7 h p.i. the luciferase expression levels were determined; (B) HeLa-CEACAM1a cells were transfected with three siRNAs directed against either GBF1, BIG1, BIG2, ARF1, firefly luciferase (luc), or GAPDH or were mock transfected (mock). Seventy-two h post transfection, the cells were inoculated with MHV-2aFLS. At 6 h p.i., the cell viability and luciferase expression levels were measured as described in the Materials and Methods. The graph depicts the relative luciferase expression (RII) compared to mock-treated cells after correction for cell viability; (C) HeLa-CEACAM1a cells were transfected with plasmids pGBF1-YFP and pARF1-YFP in the presence or absence of their corresponding siRNAs. At 24 h post transfection, the cells were fixed and the percentage of YFP-positive cells was determined; (D) HeLa-CEACAM1a cells transfected with siRNAs targeting ARF1 and mock-transfected cells were fixed at 72 h post transfection and processed for immunostaining using antibodies against the Golgi marker GM130. (E) HeLa-CEACAM1a cells were transfected with siRNAs directed against GBF1 or ARF1, or were mock transfected. Seventy-two h post transfection, the cells were inoculated with MHV-nsp2GFP and at 6 h p.i. they were fixed and processed for immunofluorescence using the nsp8 antibody. (A–C) The results of a representative experiment performed in triplicate are shown. Error bars indicate standard deviations. (D–E) Representative images are shown.

To address which ARF GEFs contribute to MHV replication, we next focused on the BFA-sensitive GEFs localized in the secretory pathway, i.e. GBF1, BIG1 and BIG2. First, we studied whether coronavirus replication was affected by BFA in MDCK cells. These cells have a BFA-resistant Golgi-apparatus due to a point mutation in GBF1 (M832L; F. van Kuppeveld, unpublished results). However, the trans-Golgi network (TGN) and the endocytic organelles in MDCK cells are still sensitive to BFA [54],[55],[56]. MDCK cells stably expressing the CEACAM1a receptor (MDCK(MHVR); [57]) were inoculated with MHV-EFLM and BFA was added either during (0–1 h p.i.) or after (1–7 h p.i.) the inoculation. The results show that MHV replication was not affected by BFA treatment of the cells during either time period (Fig. 4A), pointing toward a possible involvement of the BFA-sensitive GBF1 protein in MHV replication.

To confirm that GBF1, rather than BIG1 or BIG2, is required for MHV replication, each one of these GEFs was specifically and singularly depleted by RNA interference before assaying MHV replication. For each target gene, three siRNA oligos were transfected into HeLa-CEACAM1a cells. At 72 h post transfection, the cells were infected with the luciferase-expressing MHV-2aFLS. Six h later, the number of viable cells and the luciferase expression levels were determined (Fig. S1A and S1B) as described in the Materials and Methods. In Fig. 4B the results are presented as relative luciferase expression (RII) levels, i.e. the luciferase activity expressed relative to mock-treated cells after correction for the number of viable cells. Transfection of control siRNAs targeting the housekeeping protein glyceraldehyde 3-phosphate dehydrogenase (GAPDH) did not change the RII, whereas siRNAs targeting firefly luciferase reduced the RII up to 95% (p<0.05) demonstrating the efficiency of the siRNA transfection. Importantly, down-regulation of GBF1 resulted in a drastic inhibition of RII (p<0.05) whereas siRNAs targeting BIG1 and BIG2 did not have a significant effect (Fig. 4B). Almost identical results were obtained when the three siRNA oligos for each gene were singly transfected (data not shown). In a parallel experiment, we demonstrated that the down-regulation of the major target of GBF1, ARF1, had a similar phenotypic effect on MHV replication as seen for GBF1 (Fig. 4B).

To prove the specificity of our results, we performed a series of controls. First, the specific knockdown of the respective mRNAs after siRNA transfection was confirmed by quantitative RT-PCR analysis. At 72 h after transfection of the siRNAs, the corresponding mRNA levels for BIG1, BIG2, GBF1 and ARF1 were found to be reduced by 73%, 74%, 75%, and 94%, respectively. The mRNA levels were not affected after transfection of non-corresponding siRNAs, demonstrating the specificity of the mRNA depletion (data not shown). Second, the functional knock-down of GBF1 and ARF1 at the protein level was demonstrated by co-transfection of plasmids encoding GBF1-YFP and ARF1-YFP together with either the GBF1- or ARF1-specific siRNAs, respectively. This approach was chosen because of the unavailability of specific anti-antibodies. Twenty-four h after transfection, the cells were fixed and YFP-positive cells were counted. Fig. 4C demonstrates that GBF1 and ARF1 expression are prohibited in the presence of their specific siRNAs.

Next, we analyzed whether inhibition of MHV replication after depletion of ARF1 coincided with a collapse of the Golgi complex as observed after BFA treatment. Again, HeLa-CEACAM1a cells were transfected with siRNAs targeting ARF1 and subsequently processed for immunofluorescence at 72 h post transfection using the GM130 antibody. In the ARF1 siRNA-transfected cells, the GM130 staining was indistinguishable from that in mock-treated cells (Fig. 4D) indicating that loss of ARF1 did not lead to the collapse of the Golgi into the ER. This is in complete accordance with the results of Volpicelli-Daley *et al.* [58], who demonstrated that ARF1 depletion alone is not sufficient to mimic the BFA effect on the Golgi complex, but rather requires a simultaneous depletion of ARF1 and ARF4 [58].

Having established that depletion of GBF1 or ARF1 affects MHV replication profoundly, we studied whether the formation of the MHV RCs was similarly affected. To this end, we performed a similar knock down experiment in which we transfected siRNAs targeting either ARF1 or GBF1 and subsequently infected the cells with a recombinant MHV, which expressed an additional copy of nsp2, now fused to GFP. The nsp2-GFP fusion protein co-localizes with nsp8 and provides an additional marker for the RCs (data not shown). Six hours after infection the cells were fixed and processed for immunofluorescence with the nsp8 antibody. In mock transfected cells, many GFP and nsp8 positive foci were observed, which largely co-localized (Fig. 4E). In agreement with the relative luciferase expression values shown in Fig. 4B, both in ARF1- and GBF1-depleted cells, the number and intensity of the nsp8 positive foci was reduced, similar to what had been observed in BFA-treated cells (Fig 2B). Apparently, the number of MHV RCs is reduced in these cells. Strikingly, however, it appeared that the nsp2-GFP expression was much more affected than that of nsp8 by the depletion of either ARF1 or GBF1, as hardly any GFP fluorescence could be detected. While nsp8 is expressed directly from the viral genome, the nsp2-GFP fusion protein is expressed from a subgenomic mRNA and hence replication and transcription is required for its expression. These results therefore indicate that not only fewer RCs are formed in the absence of either GBF1 or ARF1, but that these RCs are also impaired in their RNA synthesis.

In conclusion, our results demonstrate that depletion of GBF1 and ARF1 reduces MHV replication as well as the number of RCs. Furthermore, our results indicate that the RCs formed in the absence of either GBF1 or Arf1 are less active. In addition, inhibition of MHV replication is not caused by the collapse of the Golgi apparatus *per se*, as in ARF1-depleted cells virus replication is severely affected whereas the overall morphology of the Golgi complex is unaltered.

### ARF1, COPI and PLD are not recruited to the RCs

**Figure 5:**
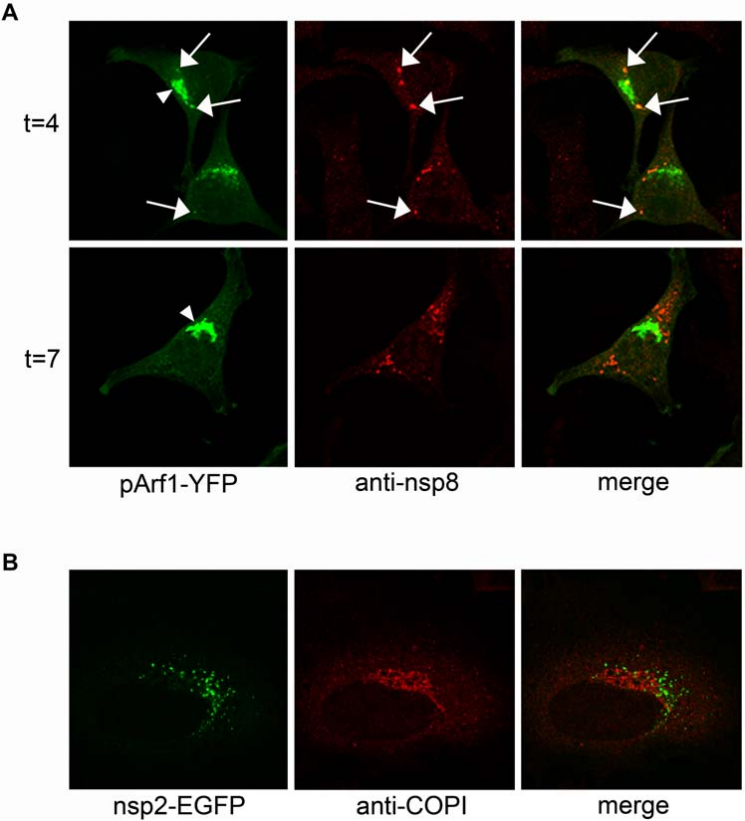
ARF1 and COPI do not co-localize with the RCs. (A) LR7 cells were transfected with pARF1-YFP, pARF1T31N-YFP, or pARF1Q71L-YFP and inoculated with MHV-A59 (moi of 1) 24 h later. At 4 h and 7 h p.i. cells were processed for immunofluorescence using antibodies against nsp8. Arrows indicate co-localization of nsp8 with ARF1; arrowheads indicate ARF1 localizing to the Golgi complex; (B) HeLa-CEACAM1a cells were inoculated with MHV-nsp2-GFP (moi of 1), fixed 7 h later and processed for immunofluorescence using antibodies against αγCOPI.

We next addressed the question whether ARF1 is recruited to the replication sites. To this end, LR7 cells expressing wild type ARF1 fused to YFP were infected with MHV-A59 and either fixed at an early (4 h) or a late (7 h) time point p.i. before identifying the replication sites by immunostaining the cells with nsp8 antibodies. Figure 5A shows that ARF1-YFP was predominantly localized to the Golgi apparatus (indicated by the arrowhead on the left panel of Fig. 5A) both at 4 h p.i. and 7 h p.i. At 4 h p.i., only in a minority of the cells co-localization between ARF1 and nsp8 was observed (indicated by the arrows in Fig. 5A). No co-localization could be observed in infected cells at 7 h p.i. Similar results were obtained for GBF1 (data not shown).

Many downstream effectors of ARF1 have been described, and the list is still growing. One of the best known functions of ARF1 involves the regulation of COPI-mediated vesicular transport. For the BFA-sensitive poliovirus, COPI has been found to localize at the replication vesicles [44]. To study whether a similar recruitment of COPI to the replication vesicles occurs during MHV replication, we determined its localization in MHV-infected cells. Thus, HeLa-CEACAM1a cells were infected with MHV-nsp2GFP. This recombinant virus allowed us to directly visualize the replication vesicles without having to perform an immunostaining with the anti-nsp8 antibodies. This was desirable as both the antibody against αγCOP (two subunits of the COPI coat) and the nsp8 antibody had been raised in rabbits. At 7 h p.i. the cells were fixed and processed for immunofluorescence analysis using the αγCOP antibody. The results show that, in addition to a diffuse staining throughout the cell, COPI was primarily localized in a Golgi-like pattern (Fig. 5B). COPI did not co-localize with the nsp2-GFP positive sites, indicating that COPI was not recruited to the replication sites of MHV.

Another well known effector of ARF1 is phospholipase D (PLD), a lipid-metabolizing enzyme involved in membrane dynamics and vesicular transport [59],[60]. To analyze whether RCs recruit PLD, LR7 cells were transfected with a construct expressing PLD1b fused to GFP and subsequently infected with MHV-A59. The cells were fixed at 7 h p.i. before identifying the replication sites by immunostaining the cells with nsp8 antibodies. No co-localization between the RCs and PLD1b could be observed (Fig. S2A). Furthermore, specific inhibition of PLD by 1-butanol [61] did not affect MHV luciferase expression compared to controls (Fig. S2B). Further studies will be required to examine the role of other ARF1 effectors.

### MHV reduces but does not block protein secretion

**Figure 6:**
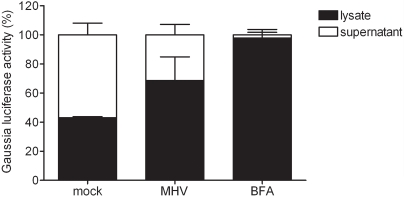
MHV reduces but does not block protein secretion. LR7 cells, transfected with a plasmid encoding the *Gaussia* gene, were at 1 h post transfection either infected with MHV-A59 or mock-infected or were treated with BFA. At 4.5 h p.i. *Gaussia* luciferase activity was determined both in the cell lysate and in the culture supernatant. The relative amount of luciferase present in the supernatant and the cell lysate is depicted.

Finally, we studied whether normal vesicular trafficking is affected in MHV-infected cells. To investigate this, we made use of a *Gaussia* reporter gene, the protein product of which is secreted upon expression [62],[63]. Cells were transfected with a plasmid encoding this gene under the control of a CMV promoter and subsequently infected with either MHV-A59, mock-infected, or treated with BFA. At 4.5 h p.i. the intracellular and extracellular levels of *Gaussia* luciferase were measured. Thus, the ratio of the luciferase activity in the cell lysate and in the culture supernatant was determined for each condition. While in mock-infected cells almost 60% of the total amount of *Gaussia* luciferase was found in the culture supernatant, in MHV-infected cells, the amount of secreted *Gaussia* luciferase was decreased about 2-fold to 30% (Fig. 6). BFA treatment inhibited, as expected, *Gaussia* protein secretion almost completely. From this we conclude that although MHV RNA replication depends on GBF1-mediated ARF1 activation, MHV infection does not drastically impair the secretory pathway. This result is not unexpected, as coronaviruses require a functional secretory pathway for the release of their progeny virions.

## Discussion

RNA viruses use and manipulate cellular membranes for the assembly of their replication and transcription structures. We and others have shown that coronaviruses exploit the early secretory pathway, but the way in which they do so is not understood. In this report we have demonstrated using several different approaches that MHV requires a functional GBF1-ARF1 pathway for efficient RNA replication. First, we showed that MHV, but not Sindbis virus replication is sensitive to BFA in murine LR7 cells. Second, we observed that MHV replication is not sensitive to BFA in MDCK cells, which contain a BFA-resistant GBF1. Third, we showed that the specific siRNA-based knockdown of the BFA-sensitive GEF GBF1, but not BIG1 and BIG2, strongly affects MHV infection. Fourth, also ARF1, a downstream effector of GBF1, appeared to be required for efficient MHV replication, as shown by the inhibition of MHV-driven reporter gene expression during siRNA-mediated down regulation of ARF1 as well as during expression of an inactive ARF1 mutant.

**Figure 7:**
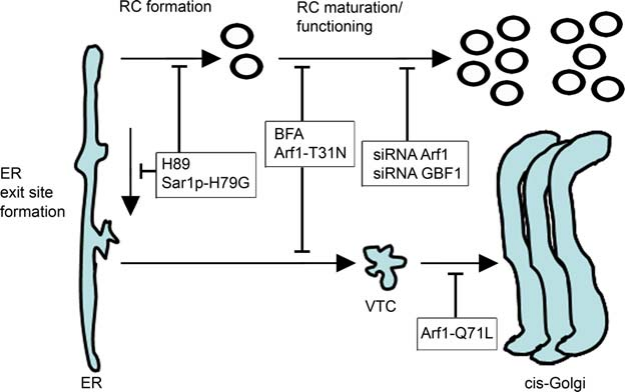
Model of MHV RCs and their links to the early secretory pathway. Two major steps in the anterograde protein secretion route (reviewed in [81]) are linked to MHV RC formation and/or RNA replication. First, transport of proteins out of the ER requires ER exit site formation controlled by Sar1p [82],[83],[84]. Blocking this early step by using the drug H89 [85] or by expressing of a dominant mutant of Sar1p [86] blocks MHV replication profoundly [11]. Next, ER exit sites develop into, or form *de novo*, vesicular-tubular clusters (VTCs) (also called ERGIC), for which GBF1 and ARF1 are required. This step, which can be blocked by BFA, by expressing a dominant-negative mutant of ARF1 or by down-regulating ARF1 using siRNAs [49], is also involved in MHV RC formation (this manuscript). However, a fully functional secretory pathway is not essential, as a dominant-active mutant of ARF1, which blocks transport between VTCs and cis-Golgi [49], does not impair MHV replication.

The inhibition of coronavirus RNA replication in the presence of BFA is either caused by direct inhibition of RC formation, resulting in reduced RNA replication, or by inhibition of RNA replication via another mechanism, resulting in reduced de novo formation of RCs. Though it is difficult to distinguish between these two scenarios, our results indicate the latter option to be most plausible. Although BFA reduced the number of RCs, their formation was not completely blocked as demonstrated by immunofluorescence staining of the RCs using the nsp8 antibody and by quantitative electron microscopy. Apparently, BFA did not prevent the formation of RCs after translation of the incoming genomic RNA. In addition, MHV replication was inhibited by BFA throughout the infection. Early in infection the inhibition was more profound than at later time points, when many transcriptionally active RCs have already been formed. Furthermore, while the inhibition of reporter gene expression in the presence of BFA, or after depletion of either GBF1 or ARF1, is in complete agreement with the reduced numbers of RCs, our results also indicate that the few RCs that are formed in the absence of GBF1 or ARF1 are less active. Therefore, we hypothesize that BFA inhibits MHV RNA replication by affecting RC maturation or functioning rather than RC formation *per se* (Fig. 7).

Replication of several viruses has now been shown to be sensitive to BFA. These viruses, which include poliovirus [39],[41],[43], grapevine fanleaf nepovirus [42] and MHV (this study), all appear to use ER-derived membranes for the formation of their RCs ([64], [42] and [10],[11],[12], respectively). Strikingly, picornaviruses belonging to different genera were found to differ in their sensitivity to BFA, which was suggested to correspond with differences in the assembly of their RCs [44]. Replication of equine arterivirus, a distant relative of coronaviruses, was observed not to be sensitive to BFA [13], while other nidoviruses have not been studied to date.

Unlike for poliovirus [65], ARF1 is hardly recruited to coronavirus RCs. We therefore hypothesize that downstream effectors of GBF1-ARF1 are involved in MHV replication. To date, more than 20 downstream effectors of ARF1 have been identified [26],[66],[67],[68], and each one of these might thus be somehow implicated in the functioning of the MHV RCs. The most well known effector of ARF1 is COPI. For picornaviruses, BFA sensitivity was suggested to correlate with the recruitment of COPI to these sites [44]. However, no co-localization between COPI and the MHV RCs could be observed. This is in agreement with the almost complete absence of ARF1 at these sites. In addition, coronavirus RCs did not co-localize with PLD1 nor was coronavirus replication affected by inhibition of phospholipase D, a lipid-metabolizing enzyme involved in membrane dynamics and vesicular transport [59],[60]. It might be that the GBF1-ARF1 pathway simply functions to deliver lipids to the RCs. In agreement herewith, cerulenin, an inhibitor of phospholipid biosynthesis, severely inhibits MHV replication (C.A.M. de Haan, unpublished results). Nonetheless, the observed inhibition of MHV infection after BFA treatment is probably not an indirect consequence of the collapse of the Golgi complex as, unlike BFA treatment, ARF1 depletion did not affect the morphology of the Golgi complex (Fig. 4D). Consistent herewith, another recent study showed that ARF1 depletion did not affect the Golgi morphology or protein transport [58].

Several studies have indicated that coronavirus replication and the ER are closely connected. Electron microscopical analyses of infected cells showed the partial co-localization of coronavirus replicase proteins with the soluble ER resident protein disulfide isomerise [10], while the DMVs were often found in close proximity to the ER and occasionally in continuous association with it [10],[12]. Furthermore, when expressed in the absence of a coronavirus infection, the nsp3 and nsp4 proteins were inserted into the ER and became modified by the addition of N-linked sugars [11],[69],[70], whereas expression of tagged MHV nsp4 in MHV-infected cells resulted in the recruitment of the protein to the replication complexes [11]. In addition, coronavirus replication was inhibited when the ER export machinery was blocked by the use of the kinase inhibitor H89 or by expression of dominant-active mutant of the small GTPase Sar1 [11]. We now show by using several approaches that MHV RNA replication also depends on GBF1-mediated ARF1 activation. Apparently, an intimate association exists between the early secretory pathway and MHV replication. Interestingly, whereas H89 blocked RC formation completely [11], this was not the case when the GBF1-mediated activation of ARF1 was impaired by BFA. Rather it appears that the RCs formed in the absence of GBF1 or ARF1 are less active, suggesting a role for these proteins in RC maturation or functioning (Fig. 7). Clearly, further investigations are needed to unravel the precise mechanism by which the secretory pathway contributes to the biogenesis of functional coronavirus RCs and to RNA replication.

## Materials and Methods

### Cells and viruses

HeLa-CEACAM1a cells were generated by transfecting HeLa cells (obtained from the MPI-CBG High-Throughput Technology Development Studio [71]) with the expression plasmid pMHVR [72] as described before [73]. Murine LR7 [74], HeLa-CEACAM1a, and Madin-Darby Canine Kidney-CEACAM1a [MDCK(MHVR); [57] cells, which all stably express the MHV receptor mCEACAM1a, were maintained as monolayer cultures in Dulbecco modified Eagle medium (DMEM; Cambrex) containing 10% fetal calf serum (FCS), 100 IU of penicillin/ml, 100 µg of streptomycin/ml (all from Life Technologies), and 0.5 mg/ml G418 (Life Technologies, Paisley, UK).

Split cells, i.e. BHK-21 cells stably expressing Sindbis virus structural proteins [75], were maintained in Glasgow MEM (Invitrogen) containing 10% FCS, 100 IU of penicillin/ml, 100 µg of streptomycin/ml, 250 µg/ml G418 and 125 µg/ml hygromycine B (Boehringer GmbH) and used to generate Sindbis pseudovirus particles containing a replicon expressing firefly luciferase. To this end, the firefly luciferase gene was cloned into the pSinRep5 vector (Invitrogen) using conventional cloning procedures. The resulting vector was subsequently processed further according to Polo *et al.* [75] to produce the pseudovirus particles.

LR7 cells were used to propagate the wild type and recombinant MHVs (based on strain A59). The recombinant viruses expressing the firefly luciferase gene (MHV-EFLM and MHV-2aFLS) or the red fluorescent protein (RFP) gene have been described before [11],[47]. The recombinant virus MHV-nsp2GFP, which expresses a nsp2-green fluorescent protein (GFP) fusion protein, was generated in a similar way as described previously for MHV-nsp4GFP [11]. Briefly, an nsp2-GFP fusion construct was cloned behind an additional transcription regulation sequence into a derivative of the RNA transcription vector pMH54 [74]. Targeted recombination to obtain the recombinant MHV-nsp2GFP was performed as described before [74].

### Antibodies and plasmids

Antibodies directed against the MHV nsp8 (anti-p22, kindly provided by M. Denison, Vanderbilt University Medical Center, Nashville, USA [51]), the amino terminus of the MHV M protein (J1.3, kindly provided by J. Fleming, University of Wisconsin, Madison, USA [76]), against αγCOPI (anti-αγCOPI, kindly provided by F. Wieland, University of Heidelberg, Germany), against GBF1 (anti-GBF1) and against the Golgi marker GM130 (anti-GM130) (the latter two from BD Transduction Laboratories, San Jose, USA) were used. The conjugated secondary antibodies were purchased from Jackson Immunoresearch Laboratories.

Plasmids containing the different ARF1 and GBF1 genes in frame with either a GFP or a yellow fluorescent protein (YFP) tag were obtained from G. Romero [77] and C. Jackson [78], respectively. pGBF1-YFP and pARF1-YFP encode the wild type proteins fused to YFP. pARF1T31N-YFP and pARF1Q71L-GFP encode a dominant-negative and a dominant-active mutant of ARF1 fused to YFP and GFP, respectively [49]. The pN-EGFP plasmid, which encodes the MHV nucleocapsid (N) protein extended at its C-terminus with GFP was constructed by cloning a PCR fragment, specifying the N gene without its stop codon, into pEGFP-N3 (Clontech), using conventional cloning procedures. The plasmid encoding the *Gaussia* reporter gene behind a CMV promoter was generated by replacing the EGFP gene in pEGFP-C1 (Clontech) with the *Gaussia* luciferase gene from pGLuc-Basic (New England Biolabs) using conventional cloning methods. The viral expression plasmid pM5f-RL-M3 was generated by cloning a synthetic DNA segment (Genscript^©^) corresponding to the extreme 5' 211 nt and the extreme 3' 401 nt of the MHV-A59 genome, separated by a *Nhe*I restriction site and flanked by a T7 promoter and a poly(A) sequence, upstream and downstream, respectively, into pUC57. Subsequently, the coding region for *Renilla* luciferase, obtained from pRLnull (Promega), was cloned into the *Nhe*I-digested vector.

### DNA transfection

Subconfluent monolayers of LR7 cells grown on coverslips in 2-cm^2^ tissue culture dishes were overlaid with transfection medium consisting of 0.2 ml of Optimem (Invitrogen) that contained 1 µl Lipofectamine 2000 (Invitrogen) and 1 µg of DNA. After 3 hours, the medium was replaced with DMEM containing 10% FCS. At 24 h after transfection the cells were processed further as indicated.

### RNA synthesis and transfection

The plasmid pM5f-RL-M3 was linearized using a *Pac*I restriction site directly downstream of the poly(A) sequence, and subsequently RNA transcripts were produced using the T7 MessageMachine Kit (Ambion) according to the manufacturer's instructions. Of the transcripts, 0.5 pmol of RNA was transfected into mock- or MHV-2aFLS-inoculated LR7 cells at 1 h p.i. using Lipofectamine 2000 (Invitrogen). Next, the cells were treated with or without 5 µg/ml BFA from 2 h until 6 h p.i., after which the cells were lysed and intracellular *Renilla* and firefly luciferase activity was measured with the Dual-Luciferase Assay Kit (Promega) according to the manufacturer's protocol.

### Confocal immunofluorescence microscopy

Cells were fixed using a 4% paraformaldehyde solution in phosphate buffered saline (PBS), and subsequently permeabilized with 0.1% Triton-X100 in PBS. Next, the cells were incubated for 1 h with the first antibody diluted in PBS containing 10% normal goat serum. After several washing steps, the cells were incubated with an appropriate dilution of secondary antibody in the same buffer for 1 h. After three subsequent washing steps, the coverslips were mounted in Fluosave (Calbiochem). The immunofluorescence staining was analyzed using a confocal laser-scanning microscope (Leica). GFP/YFP and FITC were excited at 488 nm and Cy5 at 633 nm.

### Quantification of virus replication

Virus replication was quantified by determining either the virus-driven luciferase expression levels or the amount of genomic RNA. To this end, LR7 or MDCK(MHVR) cells were inoculated at a multiplicity of infection (moi) of 1 with MHV-EFLM, MHV-2aFLS or Sindbis pseudovirus particles in the presence or absence of 5 µg/ml BFA in DMEM. After 1 h, the culture medium was replaced by DMEM containing 10% FCS and antibiotics, again in the presence or absence of 5 µg/ml BFA. At the indicated time points, the luciferase expression in the cells was determined using the firefly luciferase assay system (Promega) according to manufacturer's instructions and using a single-tube luminometer (Turner Designs, TD-20/20). Alternatively, RNA was isolated from the cells using the Qiagen RNeasy kit (Qiagen) according to the manufacturer's protocol. TaqMan single-tube reverse transcription-PCR (RT-PCR) assay (PE Biosystems, Foster City, California, USA) was performed essentially as described by de Haan *et al.* [79]. The reactions were performed in triplicate according to the manufacturer's instructions by using the TaqMan RT-PCR kit (PE Biosystems) and an ABI Prism 7700 sequence detector.

### Small interfering (si) RNA-mediated knockdown experiments

siRNA duplexes targeting different sites within the coding sequences of GBF1, BIG1, BIG2, and ARF1 were designed by and obtained from Ambion Inc. (three siRNAs per gene; nucleotide sequences available on request). siRNAs targeting GAPDH, luciferase GL2+GL3, and Kif11 (all from Ambion) were taken along as controls in each experiment. One day after seeding the HeLa-CEACAM1a cells, they were transfected with a final concentration of 10 nM siRNA using Oligofectamine (Invitrogen). Seventy-two h after transfection, the cells were inoculated with MHV-2aFLS at such a moi that approximately 10% of the mock-treated cells became infected. At 6 h post infection (p.i.), the cell number and viability was measured by Wst-1 assay according to the manufacturer's protocol (Roche Diagnostics GmbH). Subsequently, the medium was replaced by DMEM lacking phenol red (Cambrex) and Steadylite HTS firefly luciferase substrate (Perkin Elmer) was added. Luciferase expression was determined using a luminescence plate reader (Berthold Centro LB 960). Each siRNA experiment was performed in triplicate. For each well, luciferase values were corrected for the cell number and viability as determined by the Wst1 assay relative to the mock-treated cells.

To validate the functional knockdown of the targeted genes, mRNA levels of each gene were determined after siRNA transfection using Taqman Gene Expression Assays (Applied Biosystems, CA, USA), according to the manufacturer's protocol.

### ARF1/GBF1 expression assay

To determine whether siRNAs targeting the ARF1 and GBF1 genes effectively depleted HeLa-CEACAM1a cells from the corresponding proteins, a siRNA transfection experiment was performed in which 40 ng of the plasmids encoding either ARF1-YFP or GBF1-YFP were added to the transfection mixture containing the corresponding siRNAs. Twenty-four h after transfection, the cells were fixed and representative images were taken by an automated CellWorxTM microscope (Applied Precision) with a 10× objective.

### Flow cytometry

LR7 cells transfected with pARF1-YFP, pARF1T31N-YFP, or pARF1Q71L-GFP were inoculated with MHV-RFP (moi of 5) at 24 h post transfection. Two h p.i. 1 µM HR2 peptide [80] was added to inhibit syncytia formation. At 18 h p.i., the cells were collected and fixed using a 3% paraformaldehyde solution. After two washes with PBS, the samples were analyzed employing a FACScalibur™ flow cytometer (Becton Dickinson) gating for YFP/GFP-positive cells in the forward and side scatter, such that a limited cell population with similar ARF1 expression levels was selected. From the YFP/GFP-positive population, the fraction of cells expressing RFP was determined.

### Fixation of cells and embedding in Epon resin for electron microscopy (EM) analysis

LR7 cells infected with MHV-A59 and treated from 1 to 6 h p.i. with or without 5 µg/ml BFA were resuspended in 2% glutaraldehyde in 0.1 M cacodylate buffer (pH 7.4) for at least 2 h at room temperature (RT). This buffer was then replaced with fresh one and the fixation was continued overnight. Cells were then centrifuged, washed 3 times with the 0.1 M cacodylate buffer before being post-fixed in 1% OsO_4_, 1.5% ferrocyanide at 4°C for 60 min. Next, the cell pellet was washed 5 times with distilled water and left sit in the last wash for 30 min before being centrifuged and resuspended in warm 2% low melting point agar (Roche, Basel, Switzerland) and immediately spun down. After solidification of the agar on ice, the tip containing the cells was cut into small 1 mm^3^ blocks. These blocks were then dehydrated by immerging them into increasing amounts of ethanol (50%, 70%, 80%, 90%, 96% and 3 times 100%) by incubation on a rotatory wheel for at least 15 min at RT for each step. These amalgamations were followed by others in 1,2-propylene oxide (Merck, Haarlem, Netherlands)-Epon resin (3∶1) for 30 min, 1,2-propylene oxide -Epon resin (1∶1) for 30 min, 1,2-propylene oxide-Epon (3∶1) for 60 min and Epon resin overnight. The Epon solution was prepared by mixing 12 g of glycid ether 100, 8 g of 2-dodecenylsuccinic acid anhydride, 5 g of methylnadic anhydride and 560 ml of benzyldimethylamine (all from Serva, Heidelberg, Germany). The Epon resin was then replaced the following day with freshly made resin and the incubation continued for 4 h at RT. After centrifugation at 3000 rpm for 10 min, the Epon resin was polymerized by heating the sample at 63°C for 3 days. 65–80 nm sections were then cut using an Ultracut E ultramicrotome (Leica Microsystems) and transferred on Formvar carbon-coated copper grids. Sections were stained first with 6% uranyl acetate for 30 min at RT and then with a lead-citrate solution (80 mM lead nitrate, 120 mM sodium citrate, pH 12) for 2 min before being viewed. Analysis of EM sections was performed by using a Jeol1010 electron microscope.

### Counting and statistics of EM micrographs

DMVs were defined based on the two following morphological criteria: the typical double membrane and the presence of the previously described web-like structure in their proximity [10]. The size and the number of the DMVs in control and BFA-treated cells were determined by analyzing 60 randomly selected cell profiles. The results were statistically analyzed with the Student's t-test.

### Gene IDs

ARF1 (GeneID 375), GBF1 (GeneID 8729), BIG1 (GeneID 10565), and BIG2 (GeneID 10564).

## Supporting Information

Figure S1 The effect of depletion of Golgi-residing GEFs on MHV replication. (A–B) HeLa-CEACAM1a cells were transfected with three siRNAs directed against either GBF1, BIG1, BIG2, ARF1, firefly luciferase (luc) or GAPDH, or were mock transfected (mock). Seventy-two h post transfection, the cells were inoculated with MHV-2aFLS. At 6 h p.i., (A) the luciferase expression levels (RLU) and (B) the cell viability (relative to mock-treated cells) were measured.
(0.31 MB TIF)

Figure S2 The role of PLD in MHV replication. (A) LR7 cells were transfected with pPLD1 and inoculated with MHV-A59 (moi of 1) 24 h later. At 7 h p.i. cells were processed for immunofluorescence using antibodies against nsp8; (B) LR7 cells were inoculated with MHV-2aFLS (moi 1), and at 1 h p.i. they were either mock treated or treated with different amounts of 1-butanol or 2-butanol, as indicated. At 6 h p.i. luciferase expression was measured.
(3.24 MB TIF)
