# Supplementary figures and images for: Mouse Hepatitis Coronavirus RNA Replication Depends on GBF1-Mediated ARF1 Activation
Source: PLoS Pathog. 2008 Jun 13;4(6):e1000088. doi: 10.1371/journal.ppat.1000088 (PMC2398782; doi:10.1371/journal.ppat.1000088)

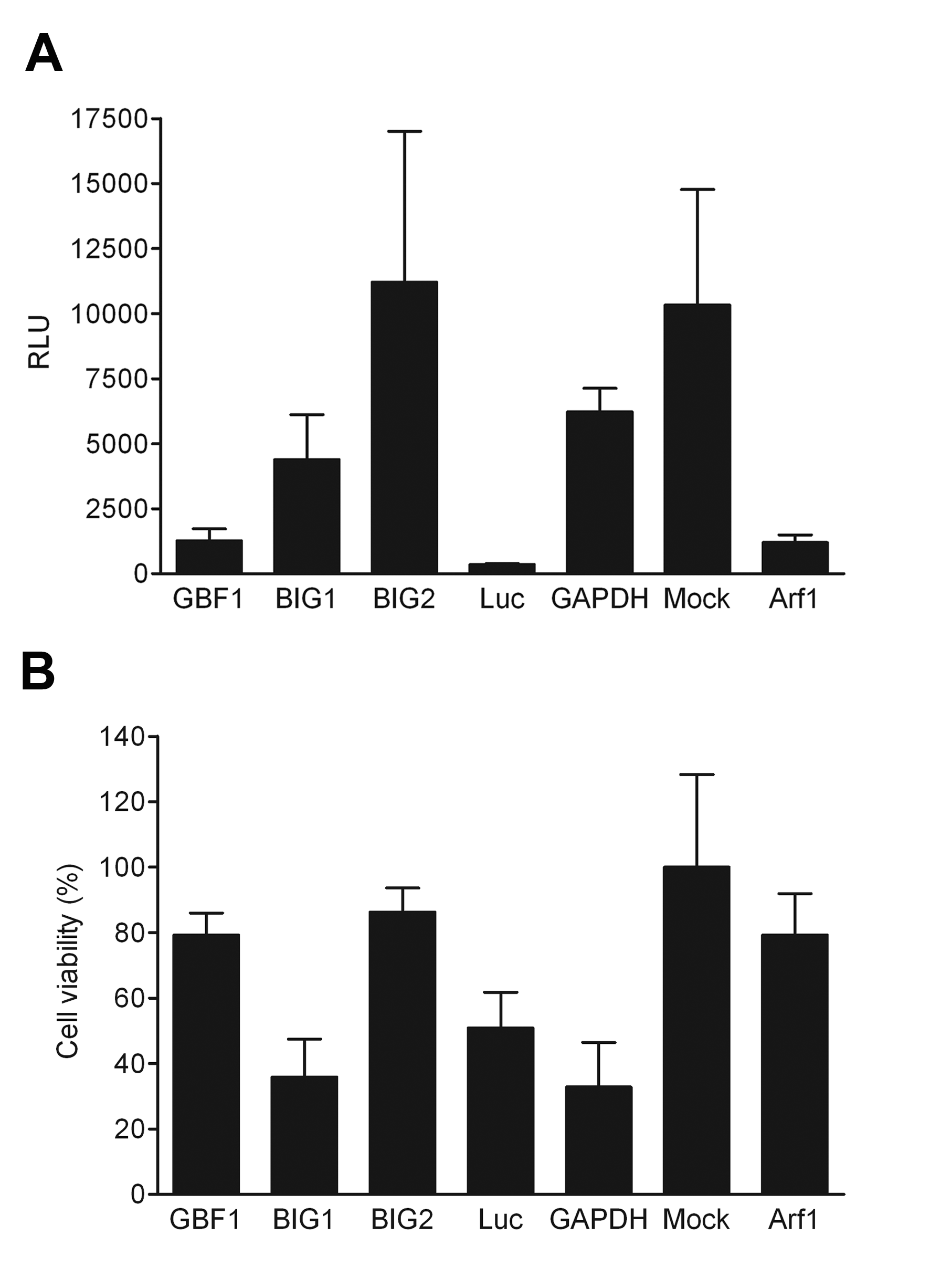

Supplement: Figure S1 — The effect of depletion of Golgi-residing GEFs on MHV replication. (A–B) HeLa-CEACAM1a cells were transfected with three siRNAs directed against either GBF1, BIG1, BIG2, ARF1, firefly luciferase (luc) or GAPDH, or were mock transfected (mock). Seventy-two h post transfection, the cells were inoculated with MHV-2aFLS. At 6 h p.i., (A) the luciferase expression levels (RLU) and (B) the cell viability (relative to mock-treated cells) were measured. (0.31 MB TIF) [file ppat.1000088.s001.tif]

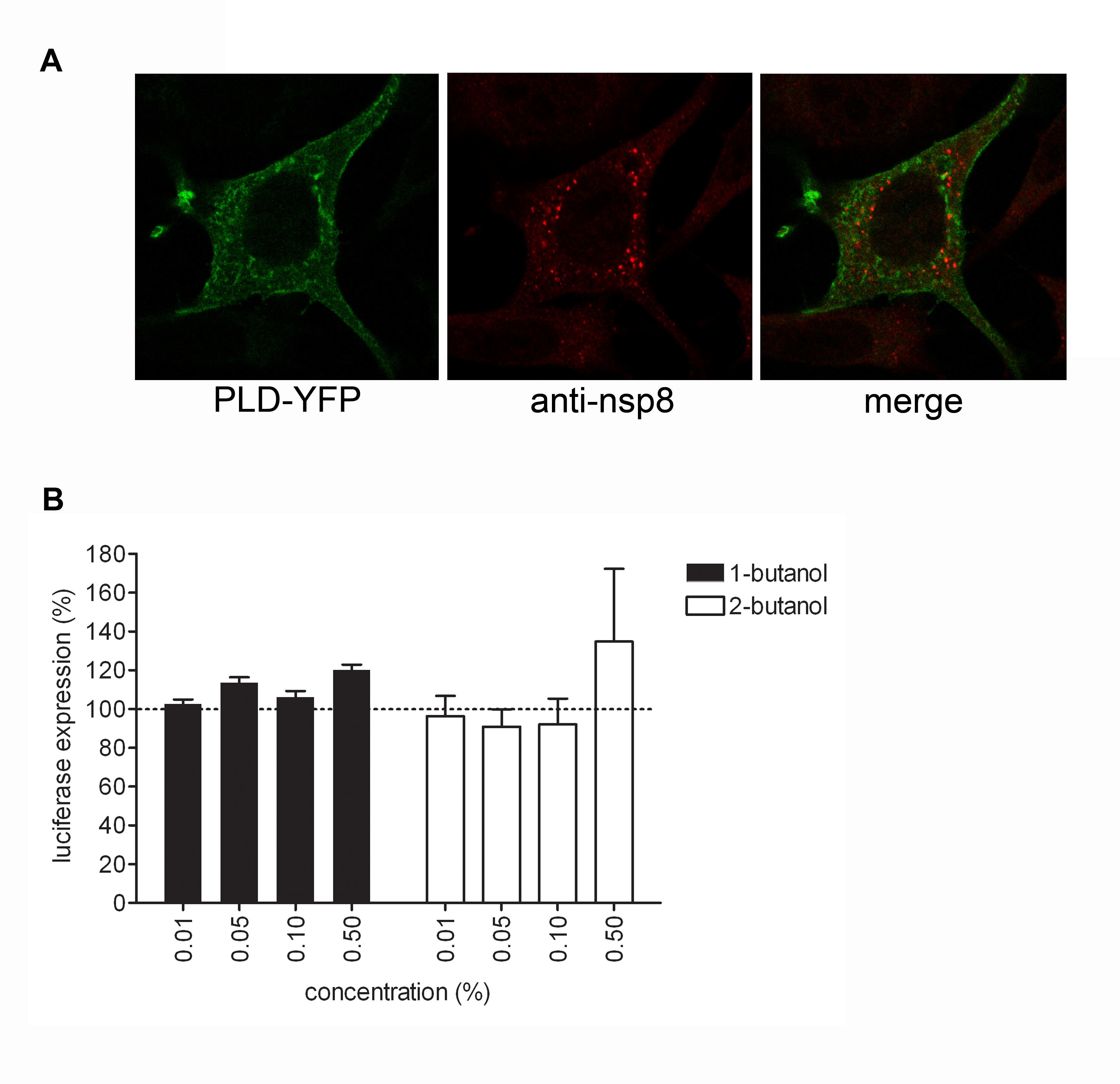

Supplement: Figure S2 — The role of PLD in MHV replication. (A) LR7 cells were transfected with pPLD1 and inoculated with MHV-A59 (moi of 1) 24 h later. At 7 h p.i. cells were processed for immunofluorescence using antibodies against nsp8; (B) LR7 cells were inoculated with MHV-2aFLS (moi 1), and at 1 h p.i. they were either mock treated or treated with different amounts of 1-butanol or 2-butanol, as indicated. At 6 h p.i. luciferase expression was measured. (3.24 MB TIF) [file ppat.1000088.s002.tif]
